# Recurrent ileocolic variceal hemorrhage treated with superselective Onyx embolization: A case report

**DOI:** 10.1016/j.radcr.2026.01.098

**Published:** 2026-03-03

**Authors:** Mohammad Reza Babaei, Fatemeh Vaezi, Bahman Rasuli

**Affiliations:** a Department of Interventional Radiology, Firouzgar Hospital, Iran University of Medical Sciences, Tehran, Iran; b Interventional Radiologist, Iran University of Medical Sciences, Tehran, Iran; c Interventional Radiologist, Arman International Hospital, Jame Jam Imaging Center, Tehran, Iran

**Keywords:** Ileocolic varices, Ectopic varices, Gastrointestinal bleeding, Onyx embolization, Interventional radiology, Portosystemic shunt

## Abstract

Ileocolic and appendicular varices are rare causes of lower gastrointestinal bleeding and are frequently missed on endoscopic evaluation. We present the case of a 35-year-old woman with ulcerative colitis, primary sclerosing cholangitis, and cirrhosis who presented with recurrent hematochezia and severe anemia. Contrast enhanced CT with venous-phase imaging revealed clusters of enhancing ileal varices, and angiography demonstrated ileocolic and appendicular varices with a significant portosystemic shunt draining into the inferior vena cava. Superselective embolization using Onyx followed by glue embolization resulted in complete cessation of bleeding. This case highlights the diagnostic value of multiphase contrast-enhanced CT including venous phase imaging and the effectiveness of minimally invasive endovascular treatment for rare ectopic varices.

## Introduction

Ectopic varices constitute less than 5% of all variceal hemorrhages and may occur anywhere along the gastrointestinal tract [1]. Ileocolic and appendicular varices are among the rarest forms and often present with recurrent or life-threatening bleeding. Because these regions are not readily visualized on endoscopy, diagnosis is frequently delayed. Cross-sectional imaging, particularly multiphase contrast-enhanced CT including venous phase, is essential for detecting abnormal variceal networks and associated venous shunts [2]. Interventional radiology plays a pivotal role in management, offering minimally invasive treatment through superselective embolization. This report presents a complex case of recurrent variceal bleeding successfully treated with Onyx embolization.

## Case presentation

A 35-year-old woman presented with recurrent hematochezia, vomiting, severe rectal bleeding, abdominal pain, progressive weakness, and weight loss. Her past medical history included biopsy-proven ulcerative colitis (2017), primary sclerosing cholangitis, cirrhosis with portal hypertension, and splenomegaly. She had undergone laparoscopic total proctocolectomy with ileoanal pouch anastomosis 4 years earlier. Family history was significant for inflammatory bowel disease in 2 cousins, one of whom died from liver failure in adolescence.

She was receiving propranolol, ursodiol, rifaximin, and levothyroxine. She reported allergies to mesalazine, octreotide, and mushrooms. Over the previous 6 months, she required multiple packed red blood cell transfusions due to gastrointestinal bleeding. Two weeks earlier, she underwent endoscopic glue injection for large fundal varices.

Laboratory findings revealed hemoglobin 6.6 g/dL, MCV 89 fL, MCH 25 pg, RDW 16%, albumin 2.6 g/dL, calcium 8.2 mg/dL, and ESR 30 mm/hr. Aside from these abnormalities, the remainder of the clinical examination and laboratory assessment was unremarkable. Endoscopy showed small esophageal varices and large fundal varices without active bleeding; colonoscopy revealed fresh blood and clots within the ileum without identifiable bleeding source.

**Fig. 1:**
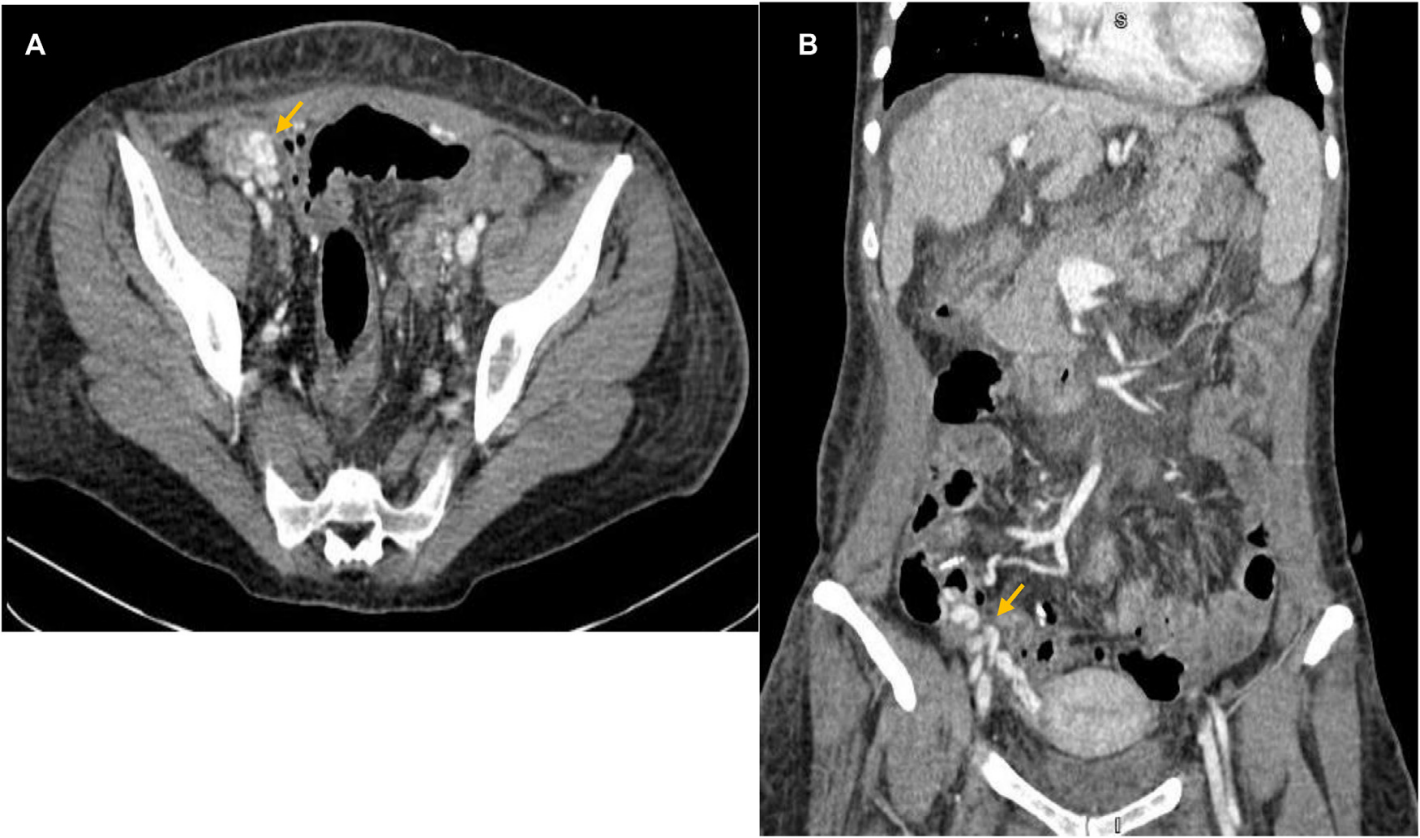
Multiphase contrast-enhanced CT images (A, B) in the venous phase (axial and coronal reconstructions) demonstrate serpiginous enhancing tubular structures (orange arrows) along the ileal loops and ileocolic territory, consistent with ectopic ileal and ileocolic varices. The largest variceal channel measures approximately 6-7 mm in diameter.

Contrast-enhanced CT of the abdomen and pelvis was performed using a multidetector CT scanner with venous-phase acquisition (slice thickness 1-1.25 mm, multiplanar reconstructions). Venous-phase images demonstrated clusters of serpiginous, enhancing tubular structures within the ileal wall and mesentery in the ileocolic region, consistent with ectopic ileal and ileocolic varices (Fig. 1). The largest variceal channel measured approximately 6-7 mm in diameter. No arterial extravasation or bowel wall ischemia was identified.

Digital subtraction angiography demonstrated vascular blush within the ileocecal territory without active contrast extravasation. Ultrasound-guided transhepatic portography was subsequently performed via a right portal vein branch using a 5-French vascular sheath. The main portal vein and intrahepatic portal branches were patent with hepatopetal flow, without evidence of focal stenosis or thrombosis. Portal pressure measurements were not obtained, as the bleeding source was suspected to originate from a localized mesenteric venous abnormality rather than diffuse portal hypertension.

**Fig. 2:**
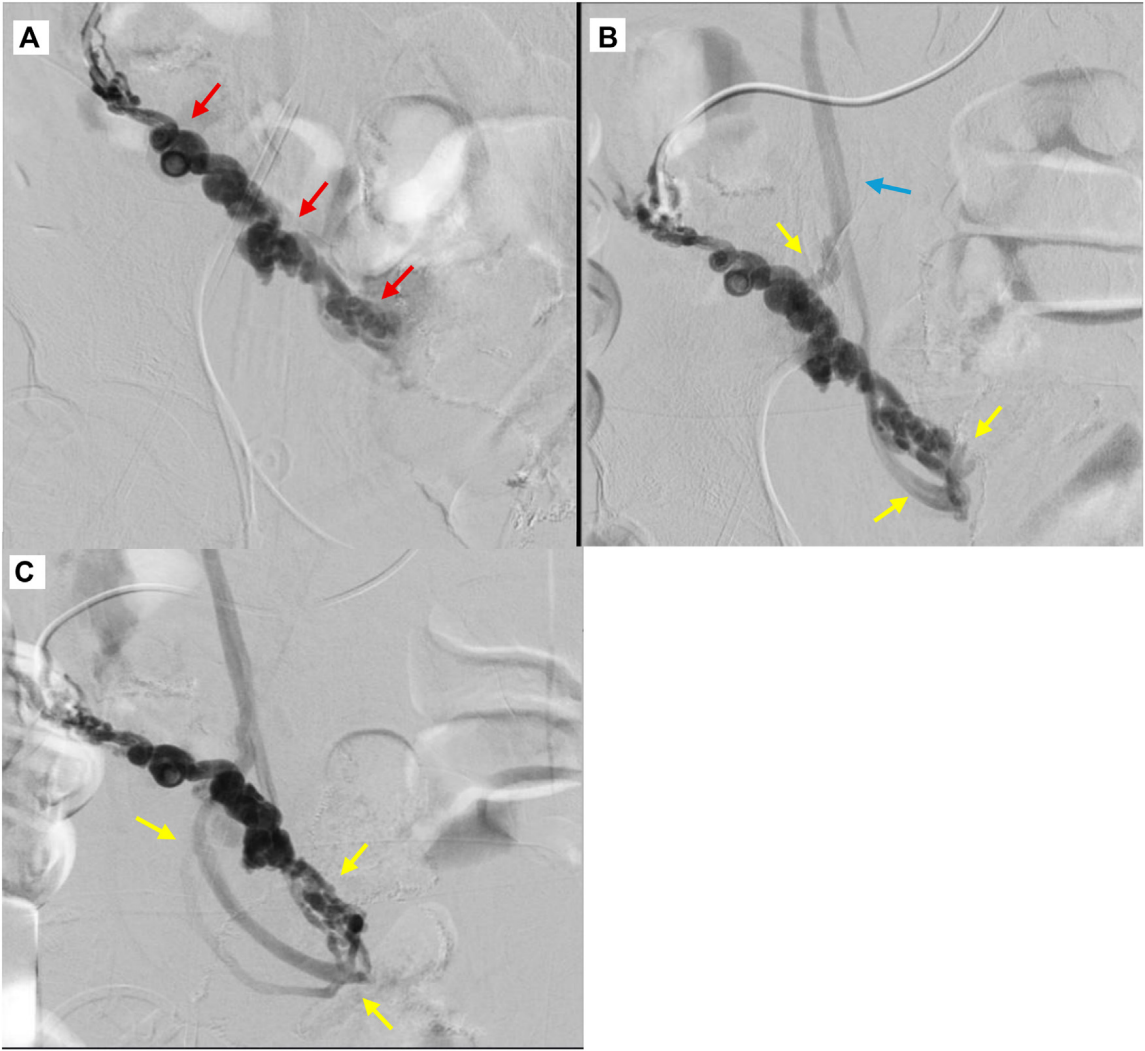
Selective superior mesenteric venography (anteroposterior projections) demonstrates (A-C) dilated serpiginous ileocolic and appendicular varices (red arrows) with a prominent high-flow portosystemic shunt (yellow arrow) draining into the inferior vena cava (blue arrow).

Selective catheterization of the superior mesenteric vein (SMV) demonstrated congested ileocolic and appendicular varices with a large high-flow portosystemic shunt draining directly into the inferior vena cava (IVC) (Fig. 2). The estimated diameter of the dominant shunt was approximately 8 mm.

Superselective embolization was performed using a 2.4-French microcatheter advanced coaxially over a 0.018-inch microwire into the draining venous channels. Given the high-flow nature of the portosystemic shunt and complex venous anatomy, Onyx 18 (Medtronic/ev3, Irvine, CA) was selected as the primary embolic agent to allow controlled injection and deep penetration while minimizing the risk of coil migration or non-target embolization. Approximately 1.5-2.0 mL of Onyx was slowly injected under continuous fluoroscopic monitoring until stable occlusion of the shunt was achieved and antegrade flow was markedly reduced.

**Fig. 3:**
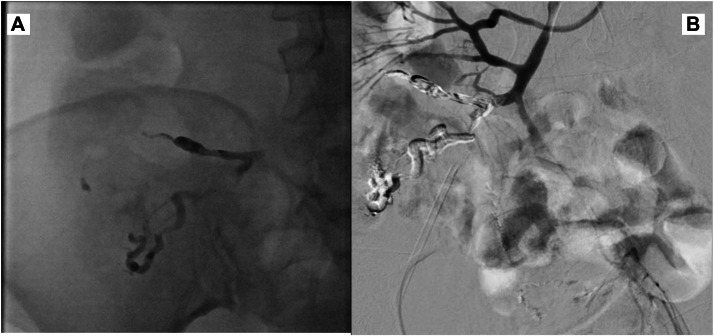
Superselective embolization using Onyx 18 followed by NBCA glue injection (A, B). Post-embolization venography demonstrates complete occlusion of the variceal complex with no residual filling.

Following successful shunt occlusion, residual tortuous variceal branches were embolized using N-butyl cyanoacrylate (NBCA) mixed with Lipiodol in a 1:3 ratio, allowing penetration into smaller collateral channels not adequately filled by Onyx alone. Care was taken to prevent reflux by slow injection and intermittent fluoroscopic pauses. Post-embolization venography demonstrated complete exclusion of the variceal complex with no residual opacification (Fig. 3).

The patient remained hemodynamically stable following the procedure. Serial abdominal examinations and laboratory monitoring showed no evidence of bowel ischemia, metabolic acidosis, or inflammatory response. No peri-procedural anticoagulation was administered, and prophylactic antibiotics were given according to institutional protocol.

The patient was discharged in stable condition with complete cessation of bleeding. At 6-month clinical follow-up, she remained asymptomatic with no recurrence of hematochezia or anemia.

## Discussion

Ectopic gastrointestinal varices represent an uncommon but clinically significant cause of gastrointestinal bleeding, accounting for approximately 1%-5% of all variceal hemorrhages [1]. These varices may arise throughout the gastrointestinal tract, including the duodenum, jejunum, ileum, colon, rectum, and postoperative anastomotic sites, and are most often associated with portal hypertension, altered mesenteric venous drainage, or surgically modified bowel anatomy [1,3]. Ileocolic and appendicular varices are among the rarest subtypes and are frequently underdiagnosed because of their deep location and limited accessibility by conventional endoscopy.

In the present case, several converging factors likely contributed to the development of complex ectopic varices, including ulcerative colitis, primary sclerosing cholangitis related cirrhosis, portal hypertension, and prior ileoanal pouch surgery. Chronic inflammatory bowel disease, particularly when associated with PSC, accelerates hepatobiliary fibrosis and can distort mesenteric venous pathways, promoting collateral formation and portosystemic shunting [[3], [4], [5], [6], [7]]. Prior colorectal surgery may further alter venous outflow patterns, increasing susceptibility to localized ectopic varices within the ileocolic and appendicular territories.

Endoscopic evaluation is frequently nondiagnostic in ileocolic and small-bowel varices due to their distal location. In contrast, contrast-enhanced CT with venous-phase imaging plays a pivotal role in detection, allowing visualization of serpiginous enhancing venous structures, variceal clusters, and associated portosystemic shunts [8]. Multiphase CT with high-resolution multiplanar reconstructions has demonstrated high sensitivity for ectopic varices and is essential for localizing the bleeding source and guiding further intervention [8]. In this case, venous-phase CT accurately identified the ileal and ileocolic varices and directed targeted angiographic evaluation.

Digital subtraction angiography remains crucial for defining venous hemodynamics and enabling definitive therapy. Selective superior mesenteric vein catheterization allows direct opacification of ileocolic and appendicular venous networks and facilitates superselective embolization in anatomically complex cases [9]. When portal flow patterns are uncertain, transhepatic portography may be useful to exclude portal vein thrombosis or focal stenosis, as performed in this patient.

Management of ectopic varices depends on the underlying etiology, venous anatomy, and patient comorbidities. Endoscopic therapy is generally ineffective for ileocolic varices and is associated with high recurrence rates [10]. Surgical resection carries substantial morbidity in patients with cirrhosis and portal hypertension and is typically reserved for refractory or anatomically isolated ectopic varices [11,12]. Contemporary surgical case literature from high-risk or resource-limited settings further emphasizes the importance of minimizing operative burden when hepatic reserve, comorbidities, or procedural risk influence treatment selection [13].

Transjugular intrahepatic portosystemic shunt effectively reduces portal pressure and is widely used for variceal bleeding; however, its efficacy may be limited when hemorrhage originates from localized mesenteric venous abnormalities rather than diffuse portal hypertension [14]. Additionally, advanced primary sclerosing cholangitis represents a relative contraindication to TIPS due to impaired hepatic reserve and increased risk of post-procedural hepatic encephalopathy [15]. Balloon-occluded retrograde transvenous obliteration techniques are well established for gastric varices but are rarely applicable to ileocolic or appendicular venous anatomy, limiting their practicality in this setting [[16], [17], [18]].

In this clinical context, superselective endovascular embolization offered a targeted and minimally invasive therapeutic option. Onyx was selected to occlude the high-flow portosystemic shunt because of its controlled delivery, slow polymerization, and ability to form a stable intravascular cast within complex venous channels, thereby reducing the risk of migration compared with coils [[19], [20], [21]]. Adjunctive NBCA embolization enabled penetration into smaller residual branches not adequately filled by Onyx alone, resulting in complete exclusion of the variceal network while minimizing non-target embolization.

This case underscores the importance of meticulous cross-sectional imaging and multidisciplinary planning in patients with atypical venous anatomy or inferior vena cava involvement, where diagnostic pitfalls and procedural risks may not be immediately apparent [22]. Although the embolization technique itself is established in contemporary interventional radiology practice, the combination of rare appendicular and ileocolic venous involvement, complex portosystemic shunt anatomy, and patient-specific contraindications to alternative therapies provides valuable educational insight for clinicians managing similar cases.

## Conclusion

Ileocolic and appendicular varices are rare causes of gastrointestinal bleeding. Multiphase contrast-enhanced CT including venous phase are essential for diagnosis, while superselective embolization offers effective and minimally invasive treatment. This case highlights the role of Onyx and glue in achieving durable hemostasis in complex ectopic variceal networks.

## Ethical compliance

Written informed consent was obtained from the patient for publication of this case and all accompanying images.

## Patient consent

The patient provided written informed consent for the publication of this case report, including all clinical data and accompanying images.
